# Increased co-expression of 4-1BB with PD-1 on CD8+ tumor-infiltrating lymphocytes is associated with improved prognosis and immunotherapy response in cervical cancer

**DOI:** 10.3389/fonc.2024.1381381

**Published:** 2024-05-02

**Authors:** Xiaonan Zhu, Yaning Feng, Peiwen Fan, Danning Dong, Jianlin Yuan, Cheng Chang, Ruozheng Wang

**Affiliations:** ^1^ The Third Department of Gynecology, Affiliated Tumor Hospital of Xinjiang Medical University, Urumqi, Xinjiang, China; ^2^ Key Laboratory of Oncology of Xinjiang Uyghur Autonomous Region, Urumqi, Xinjiang, China; ^3^ Key Laboratory of Cancer Immunotherapy and Radiotherapy, Chinese Academy of Medical Sciences, Urumqi, Xinjiang, China; ^4^ Department of Head and Neck Radiation Oncology, Affiliated Tumor Hospital of Xinjiang Medical University, Urumqi, Xinjiang, China; ^5^ Nuclear Medicine Department, Affiliated Tumor Hospital, Xinjiang Medical University, Urumqi, Xinjiang, China; ^6^ Xinjiang Uygur Autonomous Region Radiotherapy Clinical Research and Training Center, Urumqi, Xinjiang, China; ^7^ Clinical Key Specialty of the Health Commission, Urumqi, Xinjiang, China

**Keywords:** cervical cancer, 4-1BB, PD-1, co-expression, prognosis, immunotherapy efficacy

## Abstract

**Background:**

The combination of agonistic antibodies with immune checkpoint inhibitors presents a promising avenue for cancer immunotherapy. Our objective is to explore the co-expression of 4-1BB, ICOS, CD28, with PD-1 on CD8+ T cells in the peripheral blood and tumor tissue of cervical cancer(CC) patients, with a specific focus on the association between the co-expression levels of 4-1BB with PD-1 and clinical features, prognosis as well as immunotherapy response. The goal is to offer valuable insights into cervical cancer immunotherapy.

**Methods:**

In this study, 50 treatment-naive patients diagnosed with CC were enrolled. Flow cytometry was used to detect PD-1/4-1BB, PD-1/ICOS and PD-1/CD28 co-expression on CD8+ T cells. Subsequent analysis aimed to investigate the differential co-expression between peripheral blood and cancer tissue, and also the correlation between co-expression and clinical features in these patients. Gene Expression Omnibus (GEO) datasets, The Cancer Genome Atlas (TCGA) cohort, The IMvigor210 cohort, The BMS038cohort and Immunophenoscores were utilized to investigate the correlation between PD-1/4-1BB and the immune microenvironment, prognosis, immunotherapy, and drug sensitivity in cervical cancer.

**Results:**

The co-expression levels of PD-1/4-1BB, PD-1/ICOS, and PD-1/CD28 on CD8+ tumor-infiltrating lymphocytes (TILs) were significantly higher in cervical cancer patients compared to those in peripheral blood. Clinical feature analysis reveals that on CD8+ TILs, the co-expression of PD-1/4-1BB is more closely correlated with clinical characteristics compared to PD-1/ICOS, PD-1/CD28, PD-1, and 4-1BB. Pseudo-time analysis and cell communication profiling reveal close associations between the subgroups harboring 4-1BB and PD-1. The prognosis, tumor mutation burden, immune landscape, and immunotherapy response exhibit statistically significant variations between the high and low co-expression groups of PD-1/4-1BB. The high co-expression group of PD-1/4-1BB is more likely to benefit from immunotherapy.

**Conclusion:**

PD-1/4-1BB, PD-1/ICOS, and PD-1/CD28 exhibit elevated co-expression on CD8+TILs of cervical cancer, while demonstrating lower expression in circulating T cells. The co-expression patterns of PD-1/4-1BB significantly contributed to the prediction of immune cell infiltration characteristics, prognosis, and tailored immunotherapy tactics. PD-1/4-1BB exhibits potential as a target for combination immunotherapy in cervical cancer.

## 1 Introduction

Cervical cancer ranks as one of the prevalent malignancies impacting women (1), with approximately 604,000 new cases reported globally in 2020, comprising around 3.1% of all cancer diagnoses and resulting in an annual mortality of 342,000 (2). The current standard treatment approaches for cervical cancer involve surgery and radiochemotherapy. However, despite these interventions, One out of every three patients experience tumor relapse and distant spread (3). In the realm of immunotherapy, immune checkpoint inhibitors (ICIs), such as programmed cell death protein 1 (PD-1), have exhibited promising outcomes. Notably, by the close of 2021, the combination of pembrolizumab with chemotherapy ± bevacizumab received approval for first-line treatment of recurrent/metastatic cervical cancer based on Keynote-826 results. Nevertheless, the overall response rate remains modest, approximately 15% (4, 5). Suboptimal responses in some patients to ICIs may be attributed to factors such as the lack or exhaustion of tumor-infiltrating lymphocytes, undermining anti-tumor activity (6). Consequently, new therapeutic approaches and the combination of several immunomodulatory targeted regimens are required to enhance the efficacy of the present cancer immunotherapies. Co-stimulatory receptors have become a current research focus.

Studies suggest that co-stimulatory pathways are vital for the activation of T cells, and the use of Immunostimulatory antibodies targeting co-stimulatory receptors can enhance the anti-tumor capabilities of immune cells (7, 8). The majority of co-stimulatory receptors belong to the immunoglobulin superfamily (IgSF) or the tumor necrosis factor receptor superfamily (TNFRSF). Glucocorticoid-induced TNF receptor family-related protein (GITR), OX40, and 4-1BB are significant co-stimulatory receptors found in the TNFRSF, while CD28 and inducible T-cell co-stimulatory (ICOS) receptors are found in the IgSF (9). Among these, 4-1BB possesses the ability to activate exhausted T lymphocytes and exhibits strong anti-tumor effects (10, 11). Activation of the 4-1BB receptor on T cells induces cell proliferation, promotes the generation of cytokines, enhances cytotoxic potential, and augments the memory differentiation of T cells. Studies indicate that treatment with 4-1BB agonistic antibodies effectively induces anti-tumor immunity (12), and co-stimulation through 4-1BB can further potentiate the reinvigoration of T cells mediated by PD-1 blockade (13). Upon T cell activation, ICOS expression is observed, and the interaction of ICOS with its ligand, ICOSL, exhibits co-stimulatory properties fostering anti-tumor responses in Th1, CTL, and Tfh cells (14). The team led by Stephen C. Jameson found that the tissue-resident memory T cell development is promoted by the co-stimulatory molecule ICOS in tissues, leading to enhanced tumor control (15). Recent findings in murine models have revealed that the recovery of exhausted CD8+ T cells subsequent to PD-1 blockade relies on CD28. CD28 co-stimulation promotes a potent and durable PD-1+ CD8 T cell response (16). However, there is limited knowledge regarding the co-expression patterns of 4-1BB, ICOS, CD28, with PD-1 on CD8+T lymphocytes, along with their immunological and clinical significance in patients with cervical cancer.

Tumor immunotherapies that target PD-1 increase T cell responses, albeit they are frequently ineffective. Targeting co-stimulatory receptors combined with immune checkpoint inhibitors has emerged as a hopeful therapeutic approach. Here, we comprehensively analyzed the co-expression of 4-1BB, ICOS, CD28 with PD-1 on CD8+ T lymphocytes, focusing on the more closely associated PD-1 + 4-1BB co-expression in cervical cancer. We investigated the immune-related characteristics of PD-1 + 4-1BB on CD8+ T lymphocytes associated with cervical cancer, providing a theoretical basis for the combined treatment approach for cervical cancer.

## 2 Materials and methods

An illustration of the flowchart that represents the complete research may be seen in **Supplementary Figure 1**.

### 2.1 Source of specimens

Patients with treatment-naive cervical squamous cell carcinoma, who were admitted to the Affiliated Cancer Hospital of Xinjiang Medical University between November 2021 and August 2022, were included in the study. Relevant clinical data were systematically collected for analysis.

Inclusion criteria for patients were (1): histopathologically confirmed cervical squamous cell carcinoma with no prior treatment for cervical cancer at the time of enrollment; (2) Karnofsky Performance Status (KPS) score ≥80; (3) age ≥ 18 years; (4) complete serological and clinical data. Exclusion criteria were: (1) pathologically confirmed types other than squamous cell carcinoma; (2) history of any other malignancy; (3) concurrent chronic infections, infectious diseases, immune system disorders, etc.; (4) pregnant females. We enrolled a total of fifty eligible patients, with ages ranging from 30 to 82 years and a median age of 55 years. Among them, 15 were elderly individuals aged 60 and above, and 35 were 60 years or younger. Histological differentiation revealed 17 cases of low differentiation and 33 cases of moderate differentiation. Clinical staging included 9 cases of stage I, 23 cases of stage II, 16 cases of stage III, and 2 cases of stage IV. HPV16-positive cases were observed in 40 instances, with 4 other HPV-positive patients and 6 HPV-negative cases. Lymph node metastasis was present in 14 cases out of 50. Surgical interventions were performed in 31 cases, while 19 cases did not undergo surgery while 19 cases did not undergo surgery at the initial treatment. Among the patients, 17 were premenopausal, and 33 were postmenopausal. In terms of tumor infiltration depth, 7 cases had superficial infiltration (1/3), 8 had intermediate infiltration (1/3), and 16 had deep infiltration (1/3). Additionally, 20 cases presented with intravascular cancer emboli, while 13 cases had no vascular involvement. In 36 cases, the Squamous cell carcinoma antigen(SCC) was ≥1.5, and in 14 cases, it was <1.5. All patients were staged using the 2018 Federation International of Gynecology and Obstetrics(FIGO) classification. This research obtained approval from the Ethics Committee of the Affiliated Cancer Hospital of Xinjiang Medical University, and all participants provided informed consent before their inclusion.

### 2.2 Isolation of mononuclear cells from peripheral blood and tumor tissues

Ten milliliters of peripheral blood obtained from patients was anticoagulated with ethylene diamine tetraacetic acid (EDTA), followed by isolation of peripheral blood mononuclear cells (PBMCs) using density gradient centrifugation. The total cell count was determined using Trypan Blue staining. Fresh tissues (0.5 cm * 0.5 cm), acquired from treatment-naive cervical cancer patients. The samples were then dissociated using C tubes (miltenyi-biotec, catalog NO. 130-093-237) and the Human Tumor Dissociation Kit (miltenyi biotec, catalog No. 130-095-929) as per manufacturer instructions. The dissociated cells were then passed through a mesh with a pore size of 50 μm to create a solution consisting of single cells.

### 2.3 Flow cytometry

The isolated peripheral blood and tissue mononuclear lymphocytes were counted, and 6 × 10^5 cells were taken. After incubating with Live/Dead BV510 dye for 20 minutes, surface staining with antibodies was performed for an additional 20 minutes. Subsequently, 200 μL of fluorescence-activated cell sorting (FACS) fixing buffer was added to fix the cells, and flow cytometry was employed for detection. The BD LSRFortessa flow cytometer was purchased from BD in the United States, Live/Dead BV510 dye was obtained from Life Technologies in the United States, and materials including Fetal Bovine Serum (FBS), RPMI 1640 cell culture medium, human lymphocyte separation solution, Human Tumor Dissociation Kit (Miltenyi), and Phosphate Buffered Saline (PBS) were all obtained from Sigma in the United States. Monoclonal antibodies, specifically anti-human CD3-AF700, anti-human CD8-APC-H7, anti-human CD4-FITC, anti-human 4-1BB-BV786, anti-human ICOS-BV711, and anti-human CD28-PerCP-CyTM5.5, were purchased from BD in the United States.

### 2.4 Collection and processing of data

Single-cell transcriptome files of CC samples were obtained from the GEO database for datasets GSE168652 and GSE171894. Gene expression and matched survival data for 304 CC patients were downloaded using the TCGAbiolinks package, followed by normalization of the gene expression data. The 304 patients were divided into two groups according to median PD-1 and 4-1BB expression levels. This yielded a final cohort of 250 CC patients.

#### 2.4.1 Single-cell RNA statistical analysis

For cellular normalization and regression, the Seurat package (version: 4.3.0) (17) was employed. Next, the top 10 principal components were utilized to develop Uniform Manifold Approximation and Projection (UMAP) or t-SNE. We utilized a graph-based clustering methodology with a resolution of 0.5 to create unsupervised cell clusters. The clustering was performed using the top 10 main components. SingleR was used to computationally assign cell type annotations.

#### 2.4.2 Pseudo-time analysis

Single-Cell Trajectories analysis using the Monocle R package (version 2.28.0) with default parameters and DDR-Tree (18). Branch fate analysis was conducted using branch expression analysis modeling. The BEAM() function in Monocle was employed to find differential expression analysis (DEGs) along the pseudotime trajectory.

#### 2.4.3 Cell communication analysis

In order to identify possible networks of communication among clusters of cells, we utilized the CellChat R package (version 1.6.1) (19). First, we created a cellchat object employing the createCellChat() function. Next, the data was subjected to pre-processing steps including identification of overexpressed genes using identifyOverExpressedGenes(), detection of overexpressed interactions using identifyOverExpressedInteraction(), and data projection using projectData(). Following this, we computed potential communication networks using computeCommunProb, filtered communication using filterCommunication, and assessed pathway-specific communication probabilities via computeCommunProbPathway. Finally, cell-cell communication networks were aggregated via the aggregateNet function.

#### 2.4.4 CytoTRACE analysis

The relative differentiation stage of cells is predicted using Cellular Trajectory Reconstruction Analysis utilizing gene Counts and Expression (CytoTRACE) (https://cytotrace.stanford.edu/).

#### 2.4.5 SCENIC analysis

The SCENIC R package (20) was employed to infer transcription factor regulatory networks (version 1.3.1). Initially, the scenicOptions variable was constructed through initializeScenic. Subsequently, the runSCENIC function was utilized for co-expression network computation. Finally, the getAUC function was employed to obtain the Area Under the Curve (AUC) values for transcription factors across different cell types.

#### 2.4.6 Survival analysis

Messenger RNA (mRNA) data from the GEO were integrated with the TCGA database for Kaplan-Meier survival analysis.

#### 2.4.7 Analysis of the immune microenvironment

Employing the R package ‘Immune Oncology Biological Research (IOBR)’ (version 0.99.9), the Cell-type Identification By Estimating Relative Subsets Of RNA Transcripts (CIBERSORT) (21) method reevaluate the profiles of 22 immune infiltrating cells for each patient based on gene expression data. Subsequently, analysis of stromal, immune, and estimate scores between two subgroups was conducted using the ‘Estimation of STromal and Immune cells in MAlignant Tumor tissues using Expression data(ESTIMATE)’ R package (version 1.0.13) (22). We performed single-sample Gene Set Variation Analysis (ssGSVA) using the Gene Set Variation Analysis (GSVA) R package (23) to analyze tumor samples from two groups.

#### 2.4.8 Mutation analysis

Single-nucleotide variant data from TCGA were used to analyze gene mutations among the high-expression and low-expression groups. We employed the ‘Maftools’ package (24) to visualize data pertaining to somatic mutations using mutation annotation format files. A waterfall plot was used to show the top 10 genes with the highest mutation frequencies in the groups with high and low expression. Tumor Mutation Burden (TMB), defined as the somatic mutation count per megabase of the coding region, was determined by dividing the total number of mutations by the size of the specific coding region.

#### 2.4.9 Identification and functional analysis of DEGs

Differential Gene Expression Analysis with limma: Identification of DEGs with false discovery rate(FDR) < 0.05 and |log2FC| > 1, Followed by pathway enrichment analysis was performed Using Gene Ontology (GO) and Kyoto Encyclopedia of Genes and Genomes(KEGG).

### 2.5 Immunotherapy and drug sensitivity analysis

Using the ‘pRRophetic’ Package (25), pharmacological sensitivity analysis was performed. Ridge regression determined the Half-Maximal Inhibitory Concentrations (IC50) using data from the Genomics of Drug Sensitivity in Cancer (GDSC) Database (https://www.ancerrxgene.org/). The Cancer Immunome Atlas (TCIA) database (https://tcia.at/) (26) is where the Immunophenoscores (IPS) of CC were obtained.

The metastatic urothelial tumors cohort (27) (IMvigor210) and the advanced melanoma cohort (28) (BMS038), both treated with immunotherapy, were included in the analysis. The R package “IMvigor210CoreBiologies” preprocessed the IMvigor210 data. RNA-seq data underwent filtering and normalization using the “edgeR” (29) package, followed by transformation using the “voom” function within the “limma” (30) package in R. Quantification of PD-1 + 4-1BB was conducted in the IMvigor210 and BMS038 cohorts.

### 2.6 Statistical analysis

Statistical analysis was conducted using SPSS 26.0 software. The chi-square test or Fisher’s exact probability test compared count data, while the t-test was applied for quantitative data comparisons. The rank sum test was used for non-normally distributed data. Overall survival (OS), disease specific survival(DSS) and progression free interval(PFI) were estimated via the Kaplan-Meier method, with the Log-rank test, and Cox proportional risk regression model for multi-factor analysis. R (version 3.6.3) was used for statistical analysis and visualization, with ggplot2 (version 3.3.3) utilized for visualization. Differences with p-values below 0.05 were considered statistically significant.

## 3 Results

### 3.1 PD-1/4-1BB, PD-1/ICOS, and PD-1/CD28 co-expression on CD8+ TILs in cervical cancer patients was higher than in PBMCs

**Figure 1:**
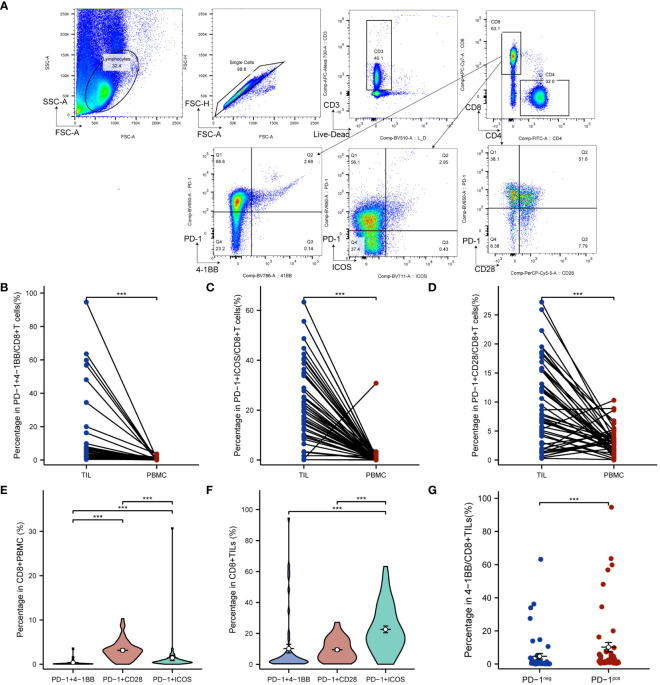
PD-1/4-1BB, PD-1/ICOS and PD-1/CD28 co-expression on CD8+ TILs in cervical cancer patients was higher than in PBMCs **(A)** Gating strategy to identify PD-1/4-1BB, PD-1/ICOS and PD-1/CD28 double positive CD8+ T cells in PBMC and TILs using flow cytometry. Comparison of PD-1/4-1BB **(B)**, PD-1/ICOS **(C)**, and PD-1/CD28 **(D)** co-expression levels in TILs and PBMCs. Comparison of PD-1/4-1BB, PD-1/ICOS, and PD-1/CD28 co-expression levels on CD8+ PBMC **(E)** and CD8+ TILs **(F)**. **(G)** 4-1BB expression according to differential PD-1 expression levels. The percentage of 4-1BB+ cells was compared among PD-1^positive^ and PD-1^neg^ subpopulations of total CD8+TILs. TILs, tumor infiltrating lymphocyte; PBMCs, peripheral blood mononuclear cells. ***p<0.001.

To investigate the co-expression levels of PD-1/4-1BB, PD-1/ICOS, and PD-1/CD28 in CC tissues and blood, a paired analysis was conducted. Flow cytometry was utilized to detect the co-expression levels of PD-1/4-1BB, PD-1/ICOS, and PD-1/CD28 on CD8+ T cells from peripheral blood and matched cancer tissues of 50 cervical squamous cell carcinoma patients (**Figure 1A**). The co-expression levels of PD-1/4-1BB on CD8+ TILs were 2.59% (interquartile range, 1.05%-6.15%), PD-1/ICOS was 19.50% (interquartile range, 11.62%-34.37%), and PD-1/CD28 was 7.45% (interquartile range, 2.94%-15.60%). On peripheral blood CD8+ T cells, the co-expression levels of PD-1/4-1BB were 0.14% (interquartile range, 0.06%-0.27%), PD-1/ICOS was 0.58% (interquartile range, 0.34%-1.05%), and PD-1/CD28 was 2.93% (interquartile range, 1.75%-3.83%). Comparative analysis revealed that the co-expression level of PD-1/4-1BB on CD8+ TILs was higher than that in PBMCs (**Figure 1B**), with a median difference of 2.37% (interquartile range, 0.76%-6.07%), (*P* < 0.001). Similarly, the co-expression level of PD-1/ICOS on CD8+ TILs was higher than that in PBMCs (**Figure 1C**), with a median difference of 18.76% (interquartile range, 11.32%-34.11%) (*P* < 0.001). Moreover, the co-expression level of PD-1/CD28 on CD8+ TILs was also higher than that in PBMCs (**Figure 1D**), with a median difference of 4.52% (interquartile range, 0.10%-12.31%) (*P* < 0.001). In CD8+ PBMCs, the co-expression level of PD-1/4-1BB was lower than that of PD-1/ICOS and PD-1/CD28 (**Figure 1E**). On CD8+TILs, the co-expression level of PD-1/4-1BB was lower than that of PD-1/ICOS (**Figure 1F**), and the differences were statistically significant. Furthermore, 4-1BB expression was lower than that of ICOS and CD28 in CD8+PBMCs and CD8+TILs (**Supplementary Figure 2**). Compared to PD-1- CD8+ TILs, 4-1BB exhibited a significant upregulation in PD-1+CD8+ TILs (**Figure 1G**).

### 3.2 Co-expression of PD-1/4-1BB on CD8+ TILs in relation to clinical prognostic features in CC patients

Based on median values, the co-expression of PD-1/4-1BB, PD-1/ICOS, and PD-1/CD28 were categorized into high and low expression groups. Clinical characteristic analysis indicated that the co-expression level of PD-1/4-1BB on CD8+ TILs correlated with lymph node spread (*P*=0.019), FIGO stage (*P*=0.041), surgical intervention status (*P*=0.016), and SCC levels (*P*=0.039). PD-1/CD28 co-expression on CD8+ TILs correlated with SCC levels (*P*=0.03), whereas PD-1/ICOS co-expression showed no association with clinical features (**Table 1**). Compared to PD-1/ICOS and PD-1/CD28, PD-1/4-1BB demonstrated a closer association with cervical cancer. Upon stratification by median values, the clinical feature analysis showed that the expression level of PD-1 in CD8+ TILs was correlated with lymph node spread (*P*=0.03) and tumor infiltration depth (*P*=0.002), while 4-1BB expression was related to surgical status (*P*=0.003) and FIGO stage (*P*=0.041) (**Supplementary Table 1**). Therefore, our focus was directed towards the co-expression of PD-1/4-1BB.

**Table 1:** Relationship between PD-1/4-1BB or PD-1/ICOS or PD-1/CD28 co-expression on CD8+ TILs and clinical features in 50 patient with cervical squamous cell carcinoma.

Characteristics	PD-1/4-1BB high co-expression	PD-1/4-1BB low co-expression	*P*	PD-1/ICOS high co-expression	PD-1/ICOS low co- expression	*P*	PD-1/CD28 high co- expression	PD-1/CD28 low co- expression	*P*
Age(years), N(%)			0.459			0.902			0.055
≤60	17 (34%)	18 (36%)		17 (34%)	18 (36%)		13 (26%)	22 (44%)	
>60	9 (18%)	6 (12%)		7 (14%)	8 (16%)		10 (20%)	5 (10%)	
Differentiation, N (%)			0.090			0.924			0.276
Moderate	20 (40%)	13 (26%)		16 (32%)	17 (34%)		17 (34%)	16 (32%)	
Poor	6 (12%)	11 (22%)		8 (16%)	9 (18%)		6 (12%)	11 (22%)	
Pelvic lymph nodes, N(%)			0.019			0.860			0.723
No	15 (30%)	21 (42%)		17 (34%)	19 (38%)		16 (32%)	20 (40%)	
Yes	11 (22%)	3 (6%)		7 (14%)	7 (14%)		7 (14%)	7 (14%)	
HPV before treatment, N (%)			0.136			0.610			0.415
HPV 16 Positive	18 (36%)	22 (44%)		18 (36%)	22 (44%)		18 (36%)	22 (44%)	
Non-HPV16 Positive	3 (6%)	1 (2%)		2 (4%)	2 (4%)		1 (2%)	3 (6%)	
Negative	5 (10%)	1 (2%)		4 (8%)	2 (4%)		4 (8%)	2 (4%)	
SCC-Ag (ng/ml), N (%)			0.039			0.420			0.030
<1.5	4 (8%)	10 (20%)		8 (16%)	6 (12%)		3 (6%)	11 (22%)	
≥1.5	22 (44%)	14 (28%)		16 (32%)	20 (40%)		20 (40%)	16 (32%)	
Surgery, N(%)			0.016			0.514			0.879
No	14 (28%)	5 (10%)		8 (16%)	11 (22%)		9 (18%)	10 (20%)	
Yes	12 (24%)	19 (38%)		16 (32%)	15 (30%)		14 (28%)	17 (34%)	
Infiltration Depth, N (%)			0.222			0.126			0.639
inner third	5 (16.1%)	2 (6.5%)		4 (12.9%)	3 (9.7%)		2 (6.5%)	5 (16.1%)	
middle third	2 (6.5%)	6 (19.4%)		6 (19.4%)	2 (6.5%)		3 (9.7%)	5 (16.1%)	
outer third	6 (19.4%)	10 (32.3%)		5 (16.1%)	11 (35.5%)		8 (25.8%)	8 (25.8%)	
Vascular cancer embolus, N(%)			0.472			1.000			1.000
No	7 (21.2%)	6 (18.2%)		6 (18.2%)	7 (21.2%)		6 (18.2%)	7 (21.2%)	
Yes	7 (21.2%)	13 (39.4%)		9 (27.3%)	11 (33.3%)		8 (24.2%)	12 (36.4%)	
FIGO stage, N (%)			0.041			0.234			0.485
I	3 (6%)	6 (12%)		6 (12%)	3 (6%)		4 (8%)	5 (10%)	
II	9 (18%)	14 (28%)		9 (18%)	14 (28%)		10 (20%)	13 (26%)	
III	13 (26%)	3 (6%)		9 (18%)	7 (14%)		7 (14%)	9 (18%)	
IV	1 (2%)	1 (2%)		0 (0%)	2 (4%)		2 (4%)	0 (0%)	

### 3.3 Pseudo-time analysis and cell communication profiling reveal close associations between the subgroups harboring 4-1BB and PD-1

**Figure 2:**
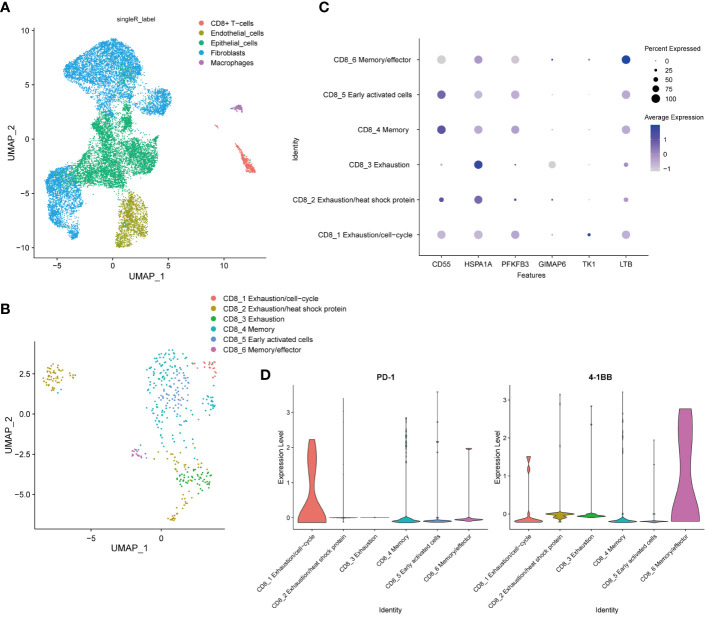
Establishment of the Single-Cell Landscape in Cervical Cancer. **(A)** UMAP view of total cells obtained from 1 CC sample in the GSE168652 dataset, color-coded by assigned cell type. **(B)** U-MAP analysis to recluster CD8+ T cells into six subpopulations based on subtype-specific gene markers. **(C)** Marker gene expression for each cell type, where dot size and color represent the percentage of the marker gene. **(D)** Violin plots showed elevated expression of PD-1 in the CD8_1 cluster and 4-1BB in the CD8_6 cluster.

To comprehensively explore the cellular composition and structure of cervical cancer, we obtained cervical cancer sample from the GSE168652 dataset in the GEO database for analysis. We constructed an atlas consisting of 15875 (tumor, 6517, normal, 9358) single cells that passed strict quality filtering. We first applied U-MAP dimensionality reduction and clustering to generate a 2D map containing 13 clusters. Using singleR annotation, we classified all cells into five distinct cell types: endothelial cells, fibroblasts, CD8+ T lymphocytes, macrophages, and epithelial cells (**Figure 2A**).

We employed U-MAP analysis to re-cluster CD8+ T cells into six clusters (**Figure 2B**). To identify the major cell subtypes, we annotated each cluster based on its marker gene expression (31). CD8+ T lymphocytes were segmented into six distinct cell clusters, including CD8_1 Exhaustion/cell-cycle (TK1), CD8_2 Exhaustion/heat shock protein (HSPA1A), CD8_3 Exhaustion (GIMAP6), CD8_4 Memory (CD55), CD8_5 Early activated cells (PFKFB3) and CD8_6 Memory/effector (LTB) (**Figure 2C**). Violin plots showed elevated expression of PD-1 in the CD8_1 cluster and 4-1BB in the CD8_6 cluster (**Figure 2D**).

**Figure 3:**
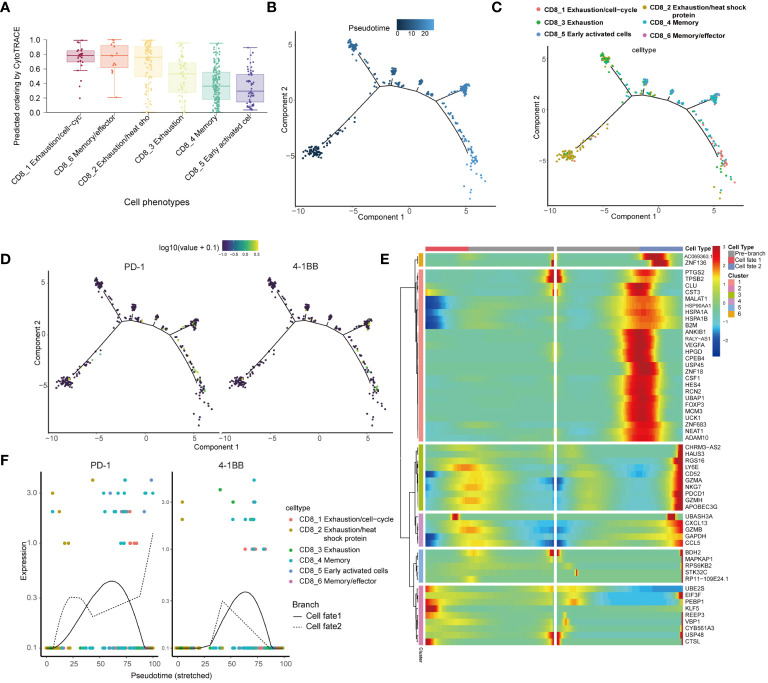
Pseudo-time analysis reveal close associations between the subgroups harboring 4-1BB and PD-1. **(A)** Boxplot showing the comparison of CytoTRACE score between different CD8 subsets. Differentiation trajectory of CD8+T cells, with each color coded for pseudo-time **(B)** and clusters **(C)**. **(D)** Fluctuations in PD-1 and 4-1BB gene expression during cell differentiation. **(E)** The differentially expressed genes (rows) along the pseudo-time (columns) were clustered hierarchically into six profiles. Color key differentially coding from blue to red indicated the relative expression levels from low to high. **(F)** Variations in the expression levels of PD-1 and 4-1BB across distinct cellular differentiation fates.

We utilized CytoTRACE to assess the differentiation potential of distinct cell subtypes. The results showed that CD8_1, CD8_6, and CD8_2 were less differentiated, while CD8_4 and CD8_5 were highly differentiated (**Figure 3A**). In order to explore the evolutionary dynamics of the CD8+ T-cell lineage in cervical cancer, we performed pseudo-temporal cell trajectory analyses of six CD8+ T-cell subpopulations. Cells of the CD8_1 population were primarily situated at the final stage of the cell trajectory, and cells of the CD8_2 population were predominantly distributed in the initial stage, with four branching points in the differentiation process (**Figures 3B, C**). Variations in PD-1 and 4-1BB expression during cell differentiation showed low expression at the initiation stage and higher expression at the terminal stage (**Figure 3D**). PD-1 expression on CD8+ T cells positively correlates with 4-1BB expression level (**Supplementary Figure 3A**). The heatmap illustrated the gene expression changes from the initial state to cell fate 1 or 2, identifying six distinct transformation patterns(**Figure 3E**), in which PD1 was also included. Enrichment analysis was performed on DEGs in various cell trajectory fates. GO analysis revealed that DEGs are mainly associated with positive regulation of response to external stimulus, positive regulation of leukocyte chemotaxis, and cytolytic granule. KEGG analysis indicated that DEGs are primarily related to Antigen processing and presentation (**Supplementary Figure 3B**). The dynamic expression patterns of PD-1 and 4-1BB are similar across different cell differentiation fates (**Figure 3F**).

**Figure 4:**
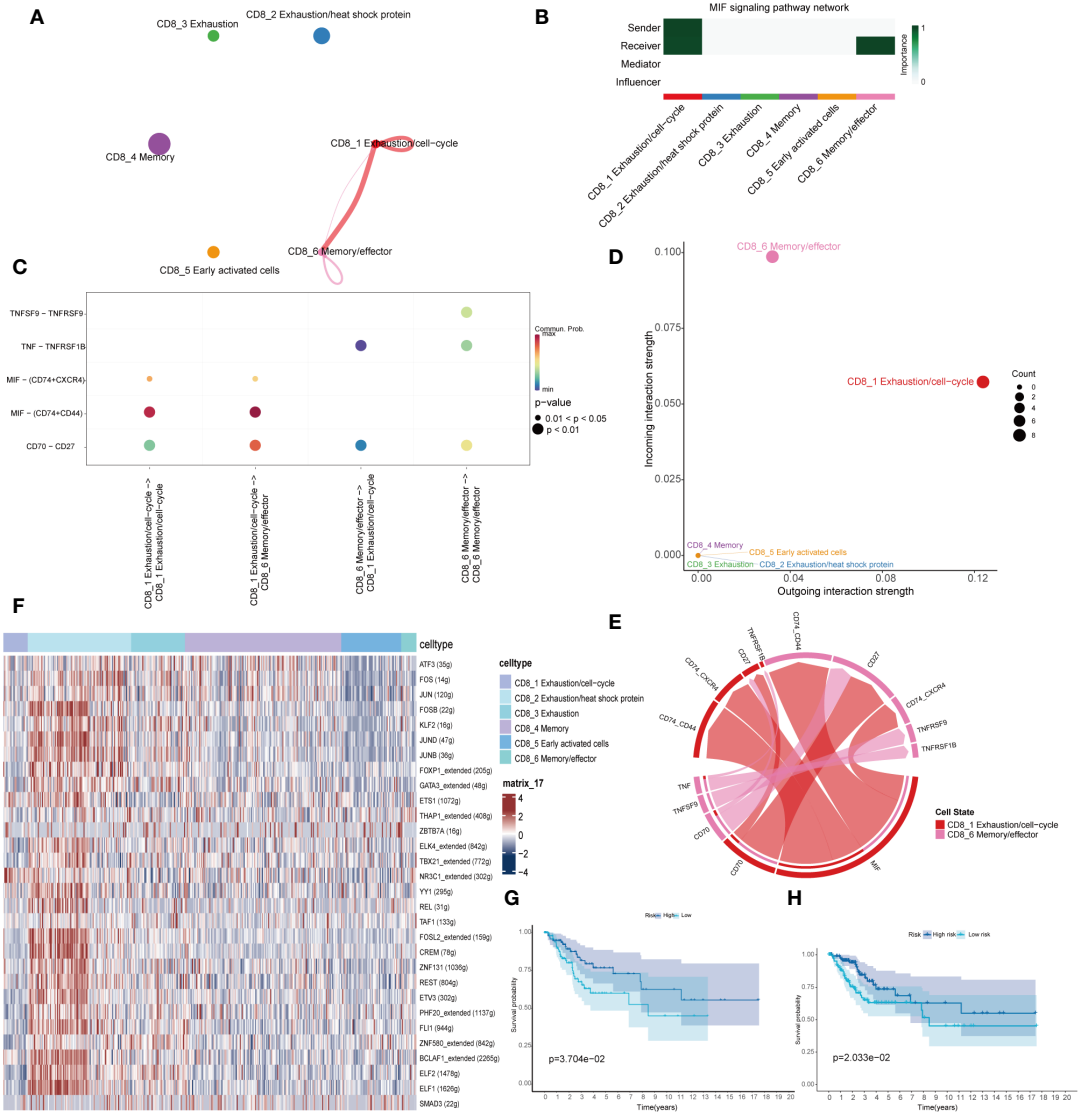
Intercellular ligand-receptor prediction among CD8+T cells and immune cells revealed by CellChat. **(A)** An overview of cell-cell interactions. **(B)** For the relative importance of each cell group based on the computed network centrality measures of signaling networks. Influencer represents a kind of cell that can control information flow within a signaling network, and a higher value indicates greater control on the information flow. The meaning of importance is the magnitude of the possibility of four roles (sender, receiver, mediator, and influencer) that the cell types play. The darker the color, the greater the role cells play. **(C)** Bubble plots of ligand-receptor pairs. Dot color reflects communication probabilities, and dot size represents computed p-values. Empty space means the communication probability is zero. p-values are computed from a two-sided permutation test. **(D)** Inferred incoming and outgoing communication patterns of CD8+T cells. The CD8_1 cluster predominantly serves as signal sender, while the CD8_6 cluster functions primarily as signal receiver. **(E)** The interplay between the CD8_1 cluster and CD8_6 cluster encompasses cellular signaling pathways and ligand-receptor interactions. **(F)** The expression of indicated transcriptional factors showed with heatmap. Combining single-cell datasets with TCGA, we performed overall survival (OS) analysis for the PD-1/4-1BB high and low co-expression groups, incorporating data from GSE168652 **(G)** and GSE171894 **(H)**.

To identify potential interactions between the six CD8+ T cells, we performed Cell Chat analysis for cell-cell signaling connections (19). Cell communication analysis revealed close interaction among the CD8_1 and CD8_6 cluster (**Figure 4A**). The CD8_1 cluster was mainly a signal sender and the CD8_6 cluster was mainly a signal receiver (**Figures 4B, D**). We examined particular ligand-receptor interactions between distinct cell subpopulations. The ligand-receptor pairs MIF-(CD74+CD44) and CD70-CD27 were significantly upregulated in the immune cell subsets CD8_1 and CD8_6 (**Figure 4C**). The pathway of macrophage migration inhibitory factor (MIF) is activated (**Figure 4E**). SCENIC analysis revealed specific activation of transcription factors NR3C1, E2F1, HOXB2 in the CD8_1 subset, and THAP1, FOXP3 in the CD8_6 subset (**Figure 4F**). The CD8_1 and CD8_6 subsets were defined as the PD-1/4-1BB high co-expression group, while the 2/4/5 subsets were defined as the PD-1/4-1BB low co-expression group. Marker genes from these two groups were selected and applied to the cervical cancer TCGA expression matrix. The expression ratio of the mean values of marker genes in the two groups was utilized to stratify them into high and low categories, followed by survival analysis. The outcomes demonstrate that the PD-1/4-1BB high co-expression group exhibits a more favorable prognosis (**Figure 4G**). Validation in the GSE171894 dataset consistently demonstrates a superior prognosis in the PD-1/4-1BB high co-expression group (**Figure 4H**).

### 3.4 Co-expression of PD-1/4-1BB is closely linked with the immune microenvironment in cervical cancer

**Figure 5:**
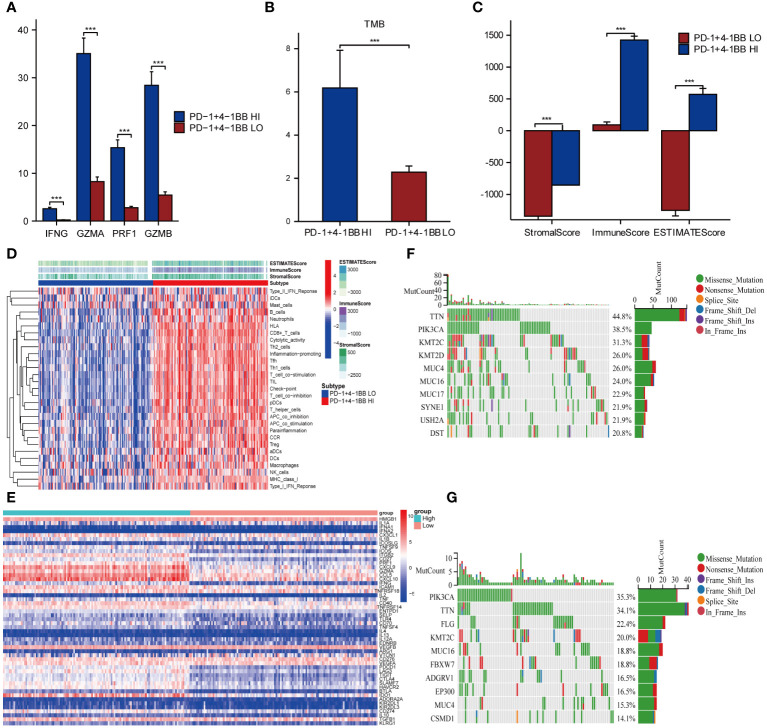
Co-expression of PD-1/4-1BB is closely linked with the immune microenvironment in cervical cancer. **(A)** Cytotoxic responses are heightened in the PD-1/4-1BB high co-expression group, featuring increased expression of (IFNG, GZMA, PRF1, GZMB). **(B)** Comparison of tumor mutation burden between high and low expression groups of PD-1/4-1BB. **(C)** Comparison of immune scores, StromaScore, ImmuneScore, and ESTIMATEScore, between the high and low expression groups. **(D)** Heatmap of the two groups based on ssGSEA scores for different immune regulatory factors. **(E)** PD-1/4-1BB high co-expression group exhibits elevated expression of both inhibitory and stimulatory immune checkpoints. **(F, G)** Waterfall plot of tumor somatic mutation in the high and low expression groups. ***p<0.001.

To explore the immune-related characteristics of PD-1/4-1BB high and low co-expression groups, we conducted an analysis assessing their correlation with immune-infiltrating cells, immune scores, immune checkpoints, cytotoxic reactions, T cell functions, and TMB. Initially, the comparative analysis of immune-related cytotoxic reactions revealed that the high co-expression group of PD-1/4-1BB exhibited stronger cytotoxic responses, including elevated levels of Interferon Gamma (IFNG), Granzyme A (GZMA), Perforin-1 (PRF1), and Granzyme B (GZMB) (**Figure 5A**). The analysis of TMB demonstrated a higher TMB among those with high expression (**Figure 5B**). Later, compared the immune scores among the PD-1/4-1BB high and low co-expression groups, revealing that the Stroma score, Immune Score, and ESTIMATE Score were all higher in the high expression group of PD-1/4-1BB (**Figure 5C**). Analysis demonstrated that the high co-expression group of PD-1/4-1BB exhibited increased expression levels of CD8 T cells, CD4 memory activated, T cells follicular helper, Dendritic cells resting, and M1 macrophages compared with the low co-expression group (**Supplementary Figure 3C**), with significant differences. The ssGSEA score heatmaps revealed greater immune infiltration in the PD-1/4-1BB high co-expression group compared to the low co-expression group (**Figure 5D**). Immune checkpoint analysis indicated that most inhibitory and stimulatory immune checkpoints had higher expression in the high co-expression PD-1/4-1BB group (**Figure 5E**). These imply a stronger association among the high co-expression group of PD-1/4-1BB and T-cell functionality. Afterwards, we conducted additional research on the distribution patterns of the top 10 somatic mutations utilizing the TCGA dataset with the aid of the “maftools” package. The most prevalent mutations were identified in TTN and PIK3A (**Figures 5F, G**).

### 3.5 Prognostic, immunotherapeutic, and drug sensitivity analyses

**Figure 6:**
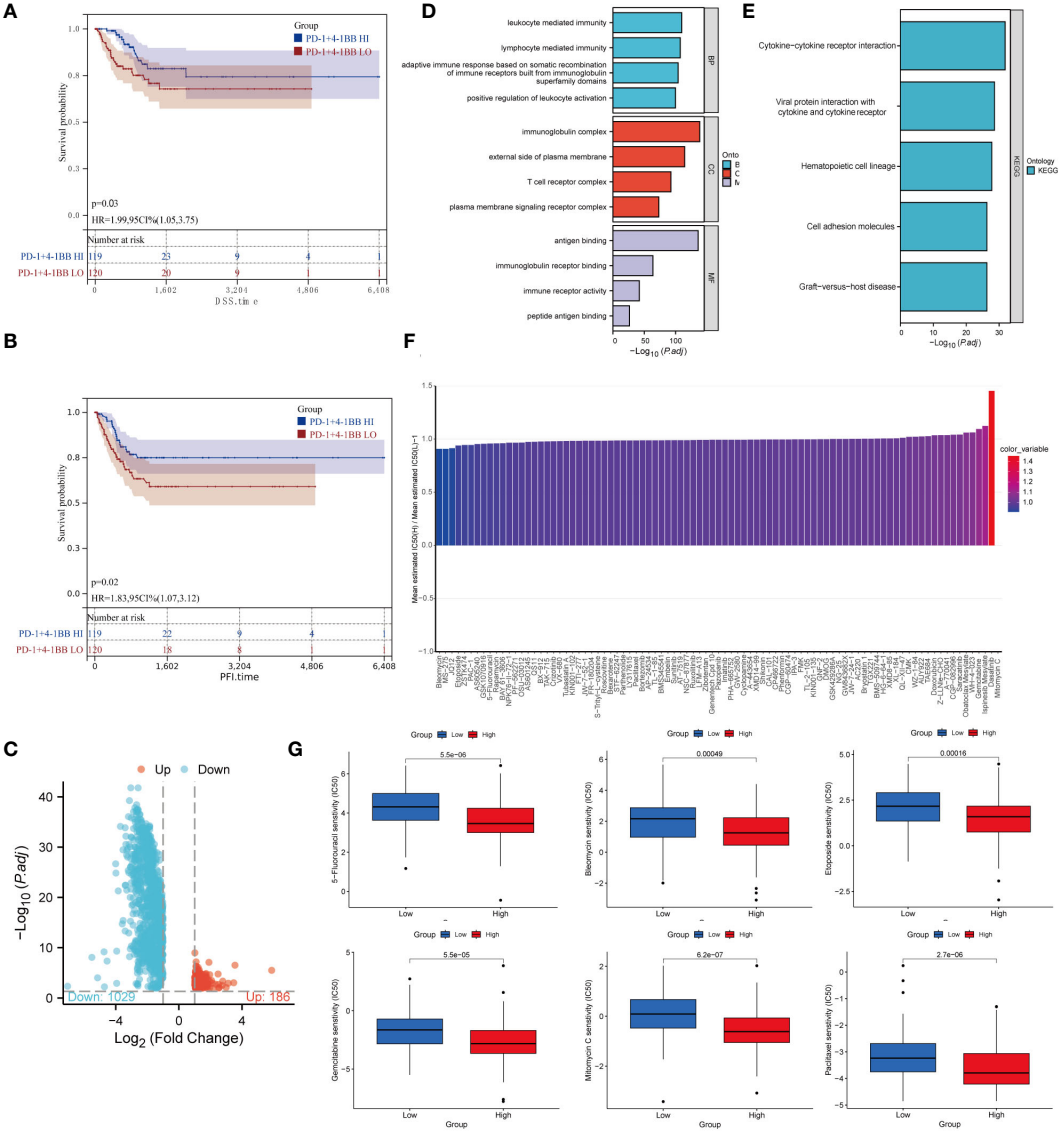
Prognostic and drug sensitivity analysis. **(A)** DSS and **(B)** PFI of patients with low or high expression groups. **(C)** Volcano plot of the distributions of all differentially expressed genes. **(D)** GO analysis; **(E)** KEGG analysis. **(F)** Stratification of PD-1/4-1BB predicts drug therapeutic benefits in CC. Proportion of normalized IC50 value of the 89 drugs between the low and high expression groups. **(G)** Comparison of IC50 values for six commonly used drugs in cervical cancer treatment, 5-Fluorouracil, Bleomycin, Etoposide, Gemcitabine, Mitomycin C, and Paclitaxel, between the high and low expression groups.

Based on the TCGA database, Kaplan-Meier analysis revealed that patients who exhibited an elevated level of co-expression of PD-1/4-1BB had improved DSS (*P*=0.03) (**Figure 6A**) and PFI (*P*=0.02) (**Figure 6B**) in comparison to the group with low co-expression, with statistically significant differences. However, no statistically significant variance emerged in terms of OS and DFI. Univariable analysis revealed that FIGO stage IV (HR 5.947, 95% CI 2.905-12.177; *P* < 0.001) and PD-1/4-1BB co-expression (HR 1.988, 95% CI 1.052-3.755; *P* = 0.034) were risk factors influencing DSS. Similarly, FIGO stage IV (HR 4.095, 95% CI 2.182-7.686; *P* < 0.001) and PD-1/4-1BB co-expression (HR 1.829, 95% CI 1.073-3.119; *P* = 0.027) were risk factors affecting PFI in univariable analysis. In multivariable analysis, FIGO stage IV (HR 5.763, 95% CI 2.820-11.815; *P* < 0.001) and PD-1/4-1BB co-expression (HR 1.953, 95% CI 1.032-3.696; *P* = 0.04) were independent prognostic factors for DSS. Moreover, FIGO stage IV (HR 3.994, 95% CI 2.126-7.504; *P* < 0.001) and PD-1/4-1BB co-expression (HR 1.860, 95% CI 1.090-3.176; *P* = 0.023) were identified as independent prognostic indicators of PFI. To explore the potential molecular mechanisms underlying the differences in DSS and PFI among the groups with high and low co-expression of PD-1/4-1BB, we conducted differential genes (**Figure 6C**) KEGG and GO enrichment analyses. GO analysis indicated enrichment of immune-related cellular functions across Biological Processes (BP), Cellular Components (CC), and Molecular Functions (MF). Differentially expressed genes in BP exhibited enrichment in lymphocyte-mediated immunity. CC analysis showed enrichment in T cell receptor complex, while MF analysis indicated enrichment in immune receptor activity (**Figure 6D**). KEGG pathway analysis demonstrated enrichment in pathways related to cytokine-cytokine receptor interaction, viral protein interaction with cytokines and cytokine receptor, hematopoietic cell lineage, and cell adhesion molecules (**Figure 6E**).

To investigate the efficacy of the high and low PD-1/4-1BB co-expression groups as indicators of how patients with CC will respond to different medicines, we calculated IC50 values for 89 drugs. Our results showed that individuals with high expression levels may demonstrate increased sensitivity to drugs such as Mitomycin C, Dasatinib, Ispinesib Mesylate, Gemcitabine, among others, while those with lower expression levels may exhibit enhanced responsiveness to Bleomycin, MS-275, and Etoposide(**Figure 6F**). Furthermore, our analysis revealed noteworthy disparities in IC50 values for six commonly employed CC therapeutics, namely Paclitaxel, Gemcitabine, Bleomycin, 5-Fluorouracil, Etoposide, and Mitomycin C (**Figure 6G**).

**Figure 7:**
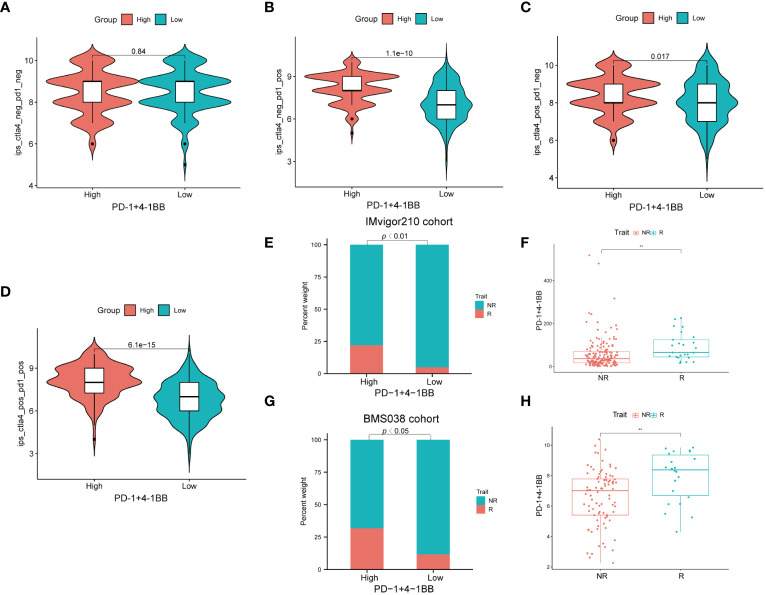
Immunotherapeutic response analysis. **(A–D)** The differences of IPS between high and low expression groups stratified by both CTLA4 and PD-1. **(E)** Proportion of patients with different treatment outcomes in high and low expression groups. The proportion of CR/PR patients in high expression group was significantly higher than that in low expression group in IMvigor210 cohort (p < 0.01). **(F)** The difference of the co-expression levels of PD-1/4-1BB between treatment outcome groups (p<0.01). The statistical difference above was compared by the Wilcoxon test. **(G)** The proportion of CR/PR patients in high expression group was significantly higher than that in low expression group in BMS038 cohort (p < 0.05). **(H)** In BMS038 cohort, The difference of the co-expression levels of PD-1/4-1BB between treatment outcome groups (p<0.01). IPS, Immunophenoscores; R=CR/PR, complete response/partial response; NR=SD/PD, stable disease/progressive disease. **p < 0.01.

Given the significance of checkpoint inhibitors in clinical practice, two approaches were employed to validate the predictive capability of PD-1/4-1BB co-expression in immunotherapeutic benefits. Utilizing the IPS, recognized as a reliable predictor of response to ICIs (26), we evaluated the effectiveness of immunotherapy. Next, we conducted a comparison of the IPS values among the cohorts with high and low expression. Elevated IPS scores are indicative of a more favorable response to ICI therapy. Our research findings reveal that in treatments involving CTLA4+/PD1−, CTLA4−/PD1+, and CTLA4+/PD1+, the high-expression cohort consistently exhibited notably higher IPS values compared to the low-expression cohort. This suggests that patients with high-expression demonstrate superior responses to anti-CTLA4, anti-PD-1, and combined anti-CTLA4 and anti-PD-1 therapies compared to those with low-expression. (**Figures 7A-D**). Compared to the low-expression group in the IMvigor210 cohort, a significantly greater percentage of patients in the high expression group achieved complete response/partial response (CR/PR) (*P <*0.01) (**Figure 7E**). Additionally, PD-1/4-1BB co-expression levels were considerably elevated in the CR/PR subgroup compared to the stable disease (SD)/progressive disease (PD) subgroup (*P* <0.01) (**Figure 7F**). Consistent with these findings, within the BMS038 cohort, a notably greater percentage of CR/PR patients was noted in the high expression group than in the low expression group (*P*< 0.05) (**Figure 7G**), with concomitantly higher co-expression levels of PD-1/4-1BB in the CR/PR subgroup compared to the SD/PD subgroup (*P*<0.01) (**Figure 7H**). Collectively, these findings reinforce the rationale for considering PD-1/4-1BB as a predictive marker of immunotherapy response. They indicate that individuals exhibiting high expression levels may stand to gain enhanced therapeutic outcomes from such interventions.

## 4 Discussion

T lymphocytes play an indispensable role in orchestrating the body’s immune response against tumors, and the distinct expression patterns and interactions between inhibitory and co-stimulatory molecules determine T cell functionality. Understanding the co-expression patterns between inhibitory receptors and co-stimulatory receptors is essential for immunological surveillance of anti-tumor immune responses (32).

4-1BB, ICOS, and CD28 are critical co-stimulatory molecules in the human body. CD28, a co-stimulatory molecule, has been identified as a major downstream target of PD-1-mediated inhibitory signaling (33, 34). Activation of the ICOS/ICOSL pathway contributes to the maintenance of T cell function in the tumor microenvironment (35). 4-1BB, a member of the TNFRSF, is a key co-stimulatory receptor. The initial phase of clinical development for the first-generation agonistic 4–1BB antibodies initiated with urelumab (BMS-663513), a humanized anti-human IgG4 antibody targeting 4-1BB. Despite initial positive outcomes, two fatal adverse events due to hepatotoxicity occurred. Further investigations demonstrated that the administration of urelumab at a safe dosage resulted in only limited efficacy (11, 36). Another monoclonal antibody called utomilumab (PF-05082566), which did not cause significant toxicities. However, it showed limited effectiveness when used alone or in combination with rituximab (11). As a result, the clinical development of this antibody was ultimately stopped. Variations exist between the two antibodies in terms of their affinity, identification of the 4-1BB receptor epitope, and isotype. The isotype also determines the Fc-gamma-receptor (FcγR) crosslinking activity (37–39). Recently, a wide range of second-generation 4–1BB agonists has been created to overcome the drawbacks of the first-generation agonists, drawing from this experience. Currently, more than 20 clinical trials are underway testing various agonistic antibodies targeting 4-1BB (40, 41). While demonstrating effective clinical responses in trials, the clinical efficacy of monotherapy remains somewhat limited. Consequently, research focus has shifted towards combinatorial strategies utilizing anti-PD-1/PD-L1 therapeutics combined with 4-1BB agonists, which have shown promise in enhancing treatment efficacy (11). In previous clinical trials of single-agent PD-1/PD-L1 blockade in recurrent or metastatic cervical cancer, reported response rates have ranged from 4% (42)to 26% (43), and disease control rates (DCR) have varied from 40% (42) to 68% (43). A recent publication reported findings from a clinical trial where heavily pretreated patients with CC received a regimen of Avelumab and Utomilumab exhibited an objective response rate (ORR) of 11% and a DCR of 78% (44). The higher DCR suggests potential additional benefits of concurrent use of 4-1BB agonists with PD-L1 blockade. Furthermore, it is worth noting that the toxicity profile associated with this combination therapy did not exceed the expectations set by monotherapy (44).

Our study reveals that the co-expression frequencies of PD-1/4-1BB, PD-1/ICOS, and PD-1/CD28 on CD8+TILs (capable of specifically recognizing and killing tumor cells) from cervical cancer are higher than those in PBMCs. In a study on liver cancer, 4-1BB exhibited prominent expression on CD8+ TILs. Particularly on cells with high PD-1 expression (13). In an ovarian cancer study, 4-1BB expression on TILs was significantly higher than in the blood (45). In a non-small cell lung cancer(NSCLC) study, PD-1/CD28 showed high co-expression in the tissue (32). These findings align with our research results. By analyzing the co-expression of three crucial co-stimulatory receptors with PD-1 and their relationship with clinical features of CC, we found that the co-expression of 4-1BB and PD-1 is more closely associated with cervical cancer. Therefore, we focus on the co-expression of PD-1/4-1BB.

Exhausted CD8+ T lymphocytes are considered the primary targets for ICI interventions (46). PD-1^high^ CD8+ TILs are indicative of highly exhausted and tumor-reactive CD8+ TILs (47, 48). Thommen et al. investigated NSCLC patients with typical T cell exhaustion phenotypic characteristics and found that PD-1^high^ expression on CD8+ TILs correlated with heightened tumor recognition capability and anti-PD-1 treatment response (48). The results demonstrated PD-1^high^ CD8+ TILs are actively involved in fighting against tumors and the exhausted phenotypes that follow. Therefore, 4-1BB expression on PD-1^high^ CD8+ TILs implies 4-1BB may selectively provide costimulatory signals to CD8+ TILs that have been actively participating in antitumor responses and highly exhausted. Thus, it is speculated that 4-1BB signaling plays a regulatory role in the exhaustion status of CD8+ T cells. In comparison to the PD-1/4-1BB low co-expression group, these cells in the high co-expression group are more tumor antigen-specific but functionally impaired. They can recover their necessary functions post-treatment, promoting tumor regression, and demonstrating broad therapeutic potential.

Single-cell RNA sequencing (scRNA-seq) represents a potent methodology for investigating the heterogeneity and dynamic changes within cellular populations. With the continuous progress of scRNA-seq, we can now delve deeper into the intricate cellular communications inside the tumor microenvironment(TME), tracing cellular proliferation and differentiation pathways, elucidating intercellular interaction networks (19, 49, 50). Within the TME, cells communicate through receptors and ligands, establishing a sophisticated signaling network associated with various behaviors, such as tumor cells growth and immune evasion (51, 52). Prospective cancer therapies might achieve efficacy by interfering with or obstructing malignancy signaling in cellular communication (53). In this study, we conducted analysis using publicly available scRNA-seq datasets and identified a mutual correlation between the cell clusters housing 4-1BB and PD-1. During the differentiation of CD8+ T lymphocytes, PD-1 and 4-1BB exhibit similar dynamic expression patterns. Analysis of signaling pathways and receptor-ligand interactions between two cell groups indicated that the MIF pathway predominantly exerts its influence. According to relevant literature, regardless of the kind of tumor, elevated levels of CD8+TILs exhibiting exhaustion markers like PD-1 before or shortly after treatment initiation are predictive of a clinical benefit from ICIs (46). Based on preliminary flow cytometry experiments, we speculate that the elevated co-expression of PD-1 and 4-1BB may be associated with prognosis or the effectiveness of immunotherapeutic interventions. Through the analysis of marker gene expression from single-cell data combined with TCGA, we have predicted a prognosis that aligns with the results obtained solely from the TCGA database, suggesting that patients exhibiting elevated PD-1/4-1BB co-expression may experience improved prognosis. Moreover, our research demonstrated that, compared to the PD-1/4-1BB low co-expression group, the high expression group was more strongly correlated with the immune microenvironment, suggesting a possible importance of PD-1/4-1BB in modulating the immune microenvironment of CC. Specifically, the PD-1/4-1BB high co-expression group exhibited more active immune responses and immune cell infiltration with cytotoxic effects, indicating a potential relevance in the advancement of new therapeutic approaches targeting the immune system in cervical cancer. In TCGA, the PD-1/4-1BB high co-expression group showed elevated expression of CD8 T cells, consistent with our experimental results. The high co-expression group of PD-1/4-1BB displayed higher immune-related scores and enhanced cytotoxic reactions compared to the low co-expression group. In a glioma study, the expression of PD-1 on 4-1BB+CD8+ TILs was reported to favorably induce higher levels of IFN-γ (54), consistent with our study findings. Functional analysis of T cells demonstrated a closer association between the PD-1/4-1BB high co-expression group and T cell-related functions compared to the low co-expression group. Additionally, this study assessed the correlation of PD-1/4-1BB with immune checkpoints and immunotherapy. According to our data, the high co-expression group had higher expression levels of immune checkpoint genes, such as cytotoxic T lymphocyte-associated antigen-4 (CTLA-4), T cell immunoreceptor with Ig and ITIM domains(TIGIT), and Lymphocyte Activation Gene-3 (LAG3). We further validated PD-1/4-1BB’s capacity for immunotherapy response using the IPS algorithm and real-world cohort. These results imply that the high co-expression subgroup might derive more advantage from immunotherapy and validate the potential of PD-1/4-1BB to predict response to immunotherapy. Our findings show that co-expression of PD-1/4-1BB can be a useful biomarker for ICI therapy in CC patients. Prior to starting treatment, PD-1/4-1BB may identify CC patients with a higher likelihood of benefiting from immunotherapy. According to recent data, CD8 T cell exhaustion phenotypes are not uniform and comprise lineage-spanning, stage-like “progenitor” and “terminally-differentiated” subtypes (55, 56). The subgroups differ in their capacity for effector function and proliferation. Progenitor exhausted CD8 T cells still have the capacity to co-produce several cytokines and undergo proliferation in the body, whereas terminally exhausted CD8 T cells are restricted to producing a single cytokine and increasing the expression of granzyme B (55, 56). Progenitor exhausted cells are less cytolytic but can survive for a longer period of time than terminally exhausted cells, which are the main cytotoxic CD8+ T cells in the TME but have a short lifespan (57). This implies that effective tumor management may involve a balance between progenitor exhausted and terminally exhausted cells (58). Progenitor exhausted CD8+ TILs demonstrate superior control over tumor growth compared to terminally exhausted T cells. Moreover, anti-PD-1 treatment can be effective for progenitor tired TILs but not for terminally exhausted TILs (55). In our study, whether high co-expression of PD-1/4-1BB is associated with alleviating T cell exhaustion or expanding the number of progenitor exhausted CD8+ T lymphocytes requires further data support.

In summary, we employed flow cytometry to explore the relationships of PD-1/ICOS, PD-1/CD28, and PD-1/4-1BB in CC tissues and blood. Comparative analysis of clinical features revealed a stronger association of PD-1/4-1BB with CC. Our study unveiled a high frequency of PD-1 + 4-1BB+CD8+ T cells, correlating with favorable prognosis. This discovery not only underscores the potential significance of this T cell subset in immunotherapy but also provides support for future personalized treatments and mechanistic investigations. Nevertheless, several limitations persist. Additional independent cohorts undergoing immunotherapy need to be examined to validate the reliability and consistency of PD-1/4-1BB as a predictor of both prognosis and immunotherapy efficacy. Further experiments are needed to investigate potential mechanisms. These findings offer valuable insights into combination immunotherapies targeting checkpoint receptors in cervical cancer.

## Data availability statement

The original contributions presented in the study are included in the article/**Supplementary Material**. Further inquiries can be directed to the corresponding author.

## Ethics statement

The studies involving humans were approved by Ethics Committee of The Afiliated Cancer Hospital of Xinjiang Medical university. The studies were conducted in accordance with the local legislation and institutional requirements. The participants provided their written informed consent to participate in this study.

## Author contributions

XZ: Data curation, Formal Analysis, Investigation, Methodology, Project administration, Software, Validation, Visualization, Writing – original draft, Writing – review & editing. YF: Data curation, Formal Analysis, Methodology, Software, Supervision, Validation, Visualization, Writing – review & editing. PF: Data curation, Formal Analysis, Investigation, Methodology, Software, Supervision, Validation, Visualization, Writing – review & editing. DD: Data curation, Formal Analysis, Methodology, Project administration, Software, Supervision, Validation, Visualization, Writing – review & editing. JY: Data curation, Investigation, Methodology, Project administration, Resources, Supervision, Validation, Visualization, Writing – review & editing. CC: Data curation, Formal Analysis, Software, Supervision, Validation, Visualization, Writing – review & editing. RW: Conceptualization, Funding acquisition, Investigation, Methodology, Project administration, Supervision, Validation, Visualization, Writing – review & editing.
