# Supplementary material for: Increased co-expression of 4-1BB with PD-1 on CD8+ tumor-infiltrating lymphocytes is associated with improved prognosis and immunotherapy response in cervical cancer
Source: Front Oncol. 2024 May 2;14:1381381. doi: 10.3389/fonc.2024.1381381 (PMC11096482; doi:10.3389/fonc.2024.1381381)
Supplement: Supplementary file 1 [file DataSheet_1.pdf]

Supplementary Material

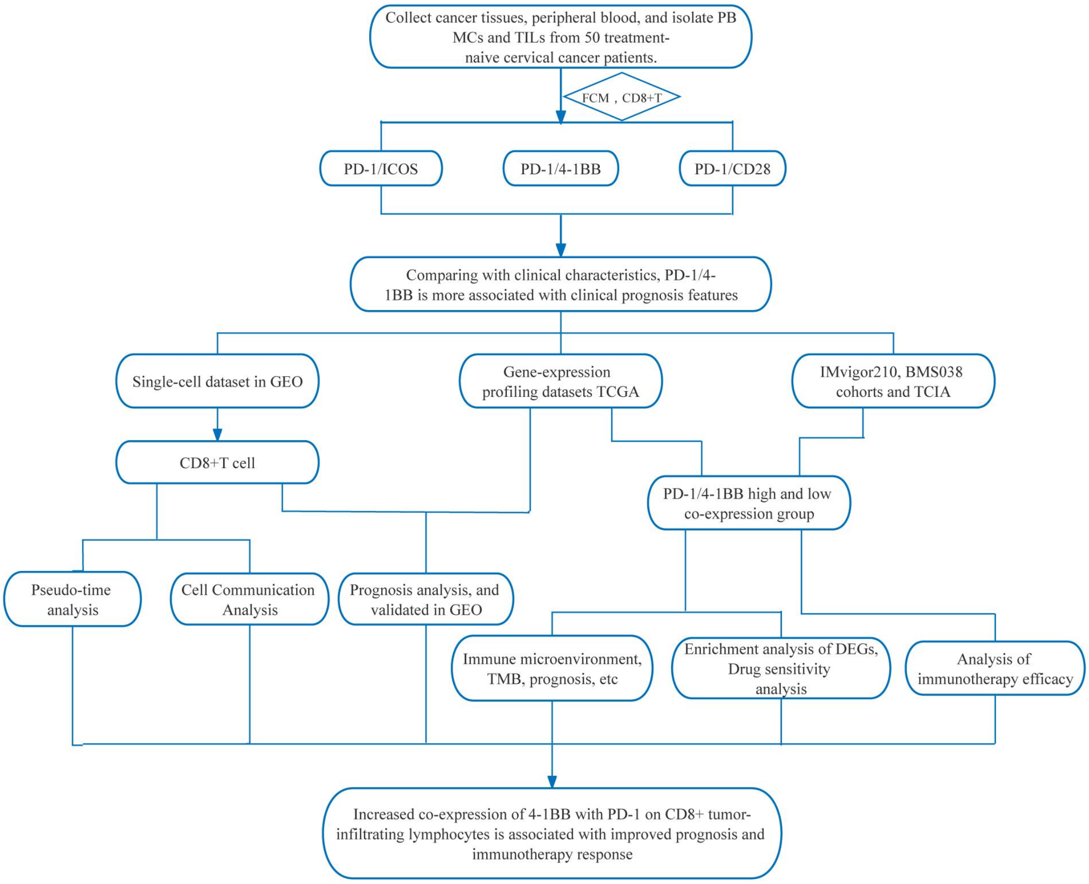

Supplementary Figure 1: The flowchart of the entire study.

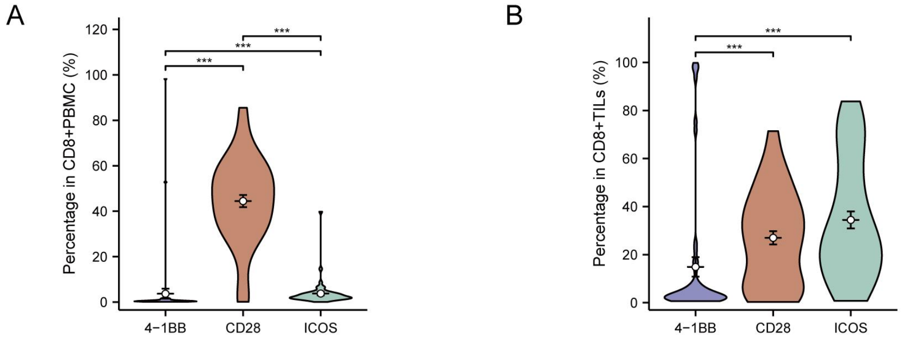

Supplementary Figure 2: A: Comparison of 4-1BB , CD28 and ICOS expression levels on CD8+ Peripheral Blood Mononuclear Cells (PBMCs) (A) and CD8+ Tumor Infiltrating Lymphocyte (TILs) (B).

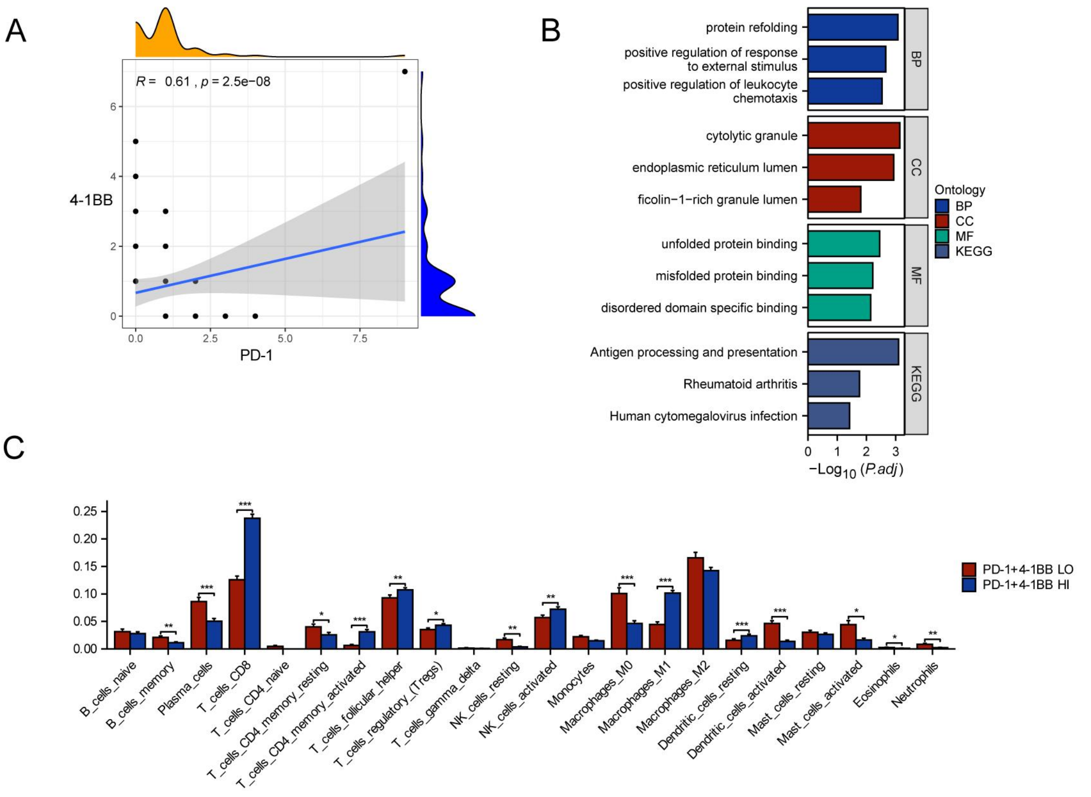

Supplementary Figure 3. (A) The scatterplots show that there was a positive correlation between PD-1 and 4-1BB. (B) Differential gene enrichment analysis in distinct cellular trajectory fates. (C) Comparison of immune-infiltrating cells between the high and low co-expression groups.

Supplementary Table 1. Relationship between 4-1BB and/or PD-1 expression on CD8+ TILs and clinical features in 50 patient with cervical squamous cell carcinoma

Characteristics	PD-1/4-1BB high co-expression	PD-1/4-1BB low co-expression	*P*	PD-1 high expression	PD-1 low expression	*P*	4-1BB high expression	4-1BB low expression	*P*
Age(years), N(%)			0.459			0.951			0.459
≤60	17 (34%)	18 (36%)		16 (32%)	19 (38%)		17 (34%)	18 (36%)	
＞60	9 (18%)	6 (12%)		7 (14%)	8 (16%)		9 (18%)	6 (12%)	
Differentiation, N (%)			0.090			0.480			0.090
Moderate	20 (40%)	13 (26%)		14 (28%)	19 (38%)		20 (40%)	13 (26%)	
Poor	6 (12%)	11 (22%)		9 (18%)	8 (16%)		6 (12%)	11 (22%)	
Pelvic lymph nodes, N(%)			0.019			0.030			0.059
No	15 (30%)	21 (42%)		20 (40%)	16 (32%)		15 (30%)	21 (42%)	
Yes	11 (22%)	3 (6%)		3 (6%)	11 (22%)		10 (20%)	4 (8%)	
HPV before treatment, N (%)			0.136			0.227			0.136
HPV 16 Positive	18 (36%)	22 (44%)		16 (32%)	24 (48%)		18 (36%)	22 (44%)	
Non-HPV16 Positive	3 (6%)	1 (2%)		3 (6%)	1 (2%)		3 (6%)	1 (2%)	
Negative	5 (10%)	1 (2%)		4 (8%)	2 (4%)		5 (10%)	1 (2%)	
SCC-Ag (ng/ml), N (%)			0.039			0.723			0.488
＜1.5	4 (8%)	10 (20%)		7 (14%)	7 (14%)		10 (20%)	7 (14%)	
≥1.5	22 (44%)	14 (28%)		16 (32%)	20 (40%)		16 (32%)	17 (34%)	
Surgery, N(%)			0.016			0.665			0.003
No	14 (28%)	5 (10%)		8 (16%)	11 (22%)		15 (30%)	4 (8%)	
Yes	12 (24%)	19 (38%)		15 (30%)	16 (32%)		11 (22%)	20 (40%)	
Infiltration Depth, N (%)			0.222			0.002			0.146
inner third	5 (16.1%)	2 (6.5%)		5 (16.1%)	2 (6.5%)		5 (16.1%)	2 (6.5%)	
middle third	2 (6.5%)	6 (19.4%)		7 (22.6%)	1 (3.2%)		2 (6.5%)	6 (19.4%)	
outer third	6 (19.4%)	10 (32.3%)		3 (9.7%)	13 (41.9%)		5 (16.1%)	11 (35.5%)	
Vascular cancer embolus, N(%)			0.472			0.296			0.717
No	7 (21.2%)	6 (18.2%)		8 (24.2%)	5 (15.2%)		6 (18.2%)	7 (21.2%)	
Yes	7 (21.2%)	13 (39.4%)		8 (24.2%)	12 (36.4%)		7 (21.2%)	13 (39.4%)	
FIGO stage, N (%)			0.041			0.415			0.041
I	3 (6%)	6 (12%)		5 (10%)	4 (8%)		3 (6%)	6 (12%)	
II	9 (18%)	14 (28%)		12 (24%)	11 (22%)		9 (18%)	14 (28%)	
III	13 (26%)	3 (6%)		6 (12%)	10 (20%)		13 (26%)	3 (6%)	
IV	1 (2%)	1 (2%)		0 (0%)	2 (4%)		1 (2%)	1 (2%)	
